# Video map: A realtime orthographic geo-image considering DEM and semantic information

**DOI:** 10.1371/journal.pone.0323669

**Published:** 2025-05-14

**Authors:** Xingguo Zhang, Xiaodi Li, Shuai Ren, Mohan Liu, Sen Yang

**Affiliations:** School of Geographic Sciences, Xinyang Normal University, Xinyang, China; Macau University of Science and Technology, MACAO

## Abstract

Aiming at the problem that it is difficult to accurately calibrate massive Pan-Tilt-Zoom Camera (PTZ) cameras on telecommunication tower and the visualization effect of orthographic geo-image is poor, this paper proposes a new method of realtime orthographic geo-image generating, which is considering Digital Elevation Model (DEM) and semantic information (ROGI-DS). First, through integrating tower cameras with 3D GIS, a camera calibration method based on view fitting (3D GIS-GeoC) is designed. Then, using the trained semantic segmentation model (TCSM), the sky area can automatically be identified and removed. Finally, based on the results of camera calibration and viewshed analysis, and the orthographic geo-image are generated. The results show that: (1) 3D GIS-GeoC method outperforms the traditional Perspective-n-Point (PnP) algorithm;(2) The tower camera semantic segmentation model (TCSM) achieves an accuracy of 96.7%; (3) ROGI-DS method improves the accuracy and visualization of orthographic geo-image under different terrain constraints, and can be used real-time monitoring of natural resources and emergency reliefs.

## 1. Introduction

With the rapid development of information and communication technology (ICT) [1], video surveillance sensors have become widely used globally and operate around the clock [2], resulting in diverse data sources and a wide range of application scenarios [3,4]. The monitoring video can record the background information of geographical scenes and the change characteristics of dynamic targets, and has the advantages of high definition, real-time and authenticity. Due to the lack of geospatial references in the video itself, integrating extensive surveillance video data with high-precision 2D and 3D geographic data, known as video GIS, has become one of the crucial methods for current geographic scene monitoring.

The surveillance video is intuitive and informative [5]. As an important way to obtain spatial-temporal information, it can accurately record the behavior characteristics [6] and patterns of each target in the geographical scene, but it lacks accurate location information [7]. The traditional methods of manual retrieval and multi-screen linkage make it difficult for managers to determine the actual orientation and motion characteristics of dynamic targets in the surveillance video, which is easy to lead to misjudgment and omission of target abnormal behavior [8]. Therefore, if the mapping between video image and geographical space is established, the static background and dynamic objects in the video can be mapped on the map. We can conduct collaborative analysis of multi camera video in geographical space, which will improve the efficiency and accuracy of intelligent video monitoring. Surveillance video is the result of real-world perspective [9], it has the characteristics of “near large and far small (the target close to the camera has a large imaging, while the target far from the camera has a small camera distance)”. so, it is not easy to measure, locate and analyze [10,11]. If the surveillance video can be orthographically processed, it can be converted into a view similar to remote sensing images, known as orthographic geo-image, which will help the deep fusion and collaborative analysis of video and geospatial data. Some scholars have explored orthographic geo-image generation, mainly including homography matrix [12], OVCM [13], etc. The former is suitable for fixed cameras and planar monitoring scenes, while the latter only removes content from non-planar regions, which is less effective in areas with terrain undulations, mainly show in stretching and deformation of non-planar regions. In addition, the semantic information of the geographic scene in the image, that is, the elements present in the image, has a significant impact on the speed and efficiency of orthographic geo-image generation.

To address the above issues, based on PTZ video, DEM, and remote sensing imagery, this paper proposes a new method of realtime orthographic geo-image generating, which is considering DEM and semantic information (ROGI-DS). Compared to existing research [13], this method focuses on PTZ cameras, which can dynamically rotate and zoom. Instead, existing homography [14] method is difficult to solve its spatial positioning problem. At the same time, the existing methods [15] are mostly used for monitoring scenes with flat terrain, and lack of video localization research in complex mountain scenes.

The paper is structured as follows: section 2 is the literature review. Section 3 describes the processing workflow and technical details of the (ROGI-DS). Section 4 analyzes the results of (ROGI-DS) based on experimental findings. Section 5 discusses the advantages, limitations and improvement direction of (ROGI-DS). Section 6 summarizes the main conclusions and describes future work plans.

## 2. Related work

Based on the technical process and related technologies in this paper, the related works is summarized from three aspects, namely camera calibration, semantic segmentation, video and GIS.

### 2.1 Camera calibration

Camera calibration is the process of determining the intrinsic, extrinsic, and distortion parameters of a camera based on its imaging model through experiments and calculations [16]. This process is crucial in measurements and positioning, 3D reconstruction [17,18], and machine vision applications [19]. Camera calibration methods can be categorized into: traditional camera calibration, active vision camera calibration, and camera self-calibration.

The traditional camera calibration method constructs camera imaging equations by using pre-set reference information as markers [20]. In 1987, Tsai [21] proposed a two-step calibration method and developed the Tsai camera imaging model. This method primarily uses the linear solution as the initial value for nonlinear optimization, achieving high accuracy. Zhang [22] proposed an algorithm for accurately calibrating a camera internal and external parameters using a planar calibration board and multi-view images. Subsequent researchers improved Zhang’s calibration method, making it applicable to a wider range of scenarios. The active vision camera calibration method refers to calibrating the camera based on known motion information of the camera. Based on the zero-distortion model, Hartley determined all five intrinsic parameters by setting the camera to perform rotational movements and capturing two images before and after the motion, which can be divided into methods based on absolute conic self-calibration, hierarchical step-by-step calibration methods, and variable intrinsic parameter camera self-calibration methods [23]. In recent years, with the rapid development of deep learning technologies, camera calibration has gradually moved towards an intelligent direction [16]. Zhang et al proposed a deep learning-based method to calibrate a PTZ camera using a pair of PTZ images [24]. This method can obtain the focal length and distortion parameters of the camera and the rotation angle between the two images; In view of the fact that the optical center of the camera does not coincide with the axis of rotation, Mathew et al [25] established the relationship between the internal and external parameters of the camera, and derived the calculation formula. Larsen [26] proposed a self- calibration method called ptcee for PTZ camera. This method can calibrate the internal and external parameters of PTZ camera in real time by combining with the directional beam adjustment (BA) measured by PTZ. However, there are still many challenges in terms of accuracy, robustness, automation level and application in dynamic environments [27].

Currently, scholars have begun to study the calibration method of PTZ camera [28–30], Chen et al. proposed a two-point calibration method for PTZ cameras, utilizing two-point correspondences and prior knowledge. However, due to the complexity and variability of natural environments [31], there is relatively limited work on precise calibration methods for multi-source heterogeneous communication tower PTZ cameras. If the EPnP [32] calibration method is employed, it demands high precision and a well-distributed set of control points, which can result in significant fieldwork efforts. Additionally, for large-scale camera networks [33], the workload for the field collection of control points is large. Therefore, there is a need for a calibration method that is automated, versatile, and does not require additional field collection of control points. In this paper, we combine tower PTZ cameras with 3D GIS and propose a geographical calibration method for using 3D-GIS (3D GIS-GeoC).

### 2.2 Semantic segmentation

Semantic segmentation is one of the classic problems in computer vision. Its core task is to assign each pixel in the input image to a predefined semantic category. In this way, each object in the image can be accurately recognized, and the scene information expressed in the image can be deeply understood [34]. Semantic segmentation can be described as the process of classifying objects within an image. For instance, given an image containing grass, a dog, and a chair, semantic segmentation assigns all pixels representing grass to one category, those representing the dog to another, and those representing the chair to a third category. Each pixel is then labeled with a distinct color corresponding to its category, allowing the computer to clearly differentiate between the various regions in the image. Furthermore, semantic information and semantic categories are related but distinct concepts. Semantic information provides a generalized description of the content within the image, emphasizing the type of information present, while semantic categories focus on dividing the image content into specific classes.

Traditional segmentation methods primarily rely on low-level features of the image itself, such as color, texture, and shape, to classify target categories. These methods are mainly used for simple scenes and tasks. In recent years, the emergence and rapid development of deep learning technologies [35] have brought revolutionary changes to the field of image segmentation. In 2015, Jonathan Long et al. introduced FCN [36,37], marking a shift in the field of image semantic segmentation from traditional methods to deep learning-based approaches. It lays a foundation for the development of more complex and efficient deep learning semantic segmentation models, such as U-Net, PSPNet, SegNet, and Deep Lab [38]. Among them, DeepLab network has high performance, flexibility and robustness, and has been widely used in the fields of urban planning and natural environment monitoring. The DeepLab includes four models: DeepLabv1 [39], DeepLabv2 [40], DeepLabv3 [41], and DeepLabv3+ [42]. Currently, there are relatively few semantic segmentation models tailored for high-altitude communication tower monitoring scenarios. At the same time, their generalization ability is relatively weak due to the complexity and variability of the natural environment. Therefore, in this paper, based on DeepLabv3 + , a large number of classification samples are collected in the experimental area, and a semantic segmentation model is constructed in the high-altitude communication tower scene by means of transfer learning. The model is used to automatically extract the sky categories in the video.

### 2.3 Video and GIS

Video surveillance, with the characteristics of low cost and real-time, can continuously record the real geographical space. However, the individual cameras have a weak sense of spatial location [43], scattered and independent. In contrast, Geographic Information Systems (GIS) provide rich spatio-temporal information through high-precision maps, offering advantages such as location and measurement capabilities and an overview [44]. However, GIS primarily presents static representations of features and struggles to provide real-time information about scenes. The combination of video and GIS, that is, Video GIS, can use the real-time monitoring video and the spatialization of geographical scenes to complement and enhance each other [45]. In 1978, Lippman [46] first combined video with geographic data to develop a dynamic and interactive map service system, which enhanced map visualization by using dynamic information obtained from real-time surveillance video. In recent years, the rapid increase in the number of video sensors has made the acquisition of various video data relatively easy [47]. Meanwhile, the rapid development of artificial intelligence technologies, represented by deep learning, has strengthened the integration of video GIS and expanded its applications [48]. In the field of urban security, Zhang [15] proposed a real-time map construction method combining multiple cameras with GIS. This method fuses targets within the overlapping fields of view of multiple cameras to provide a unified monitoring view for the geographic scene. Zhang [12] proposed a method for crowd density estimation and map visualization (CDEM-M) based on surveillance video and GIS. This method addresses the problem where existing crowd counting approaches cannot accurately count large-scale crowds or provide proper map visualization. In the field of intelligent transportation, Luo [49] proposed a 3W1H traffic violation analysis method from a GIS-geospatial perspective, which can detection of traffic violations in a geospatial context.

At present, the research of video GIS is gradually expanding from the field of urban space to the field of natural resources. Wang [50] developed a deep learning-based audio rainfall benchmark model, which can accurately estimate rainfall intensity from surveillance audio. However, existing research often targets urban and transportation scenes, video geo-mapping primarily using homography methods, which maps the location of intelligently perceived objects, such as people and vehicles, to the two-dimensional map, and pays less attention to the dynamic and real-time geolocation methods of PTZ video. At the same time, less attention is paid to the context in the video, while the contextual information is particularly important for the field of natural resource monitoring. Therefore, this paper combines the tower camera with 3D GIS, constructs a mutual mapping model of the two, and based on these models, this paper proposes a new method of realtime orthographic geo-image generating, which is considering DEM and semantic information (ROGI-DS).

## 3. Methodology

**Fig 1:**
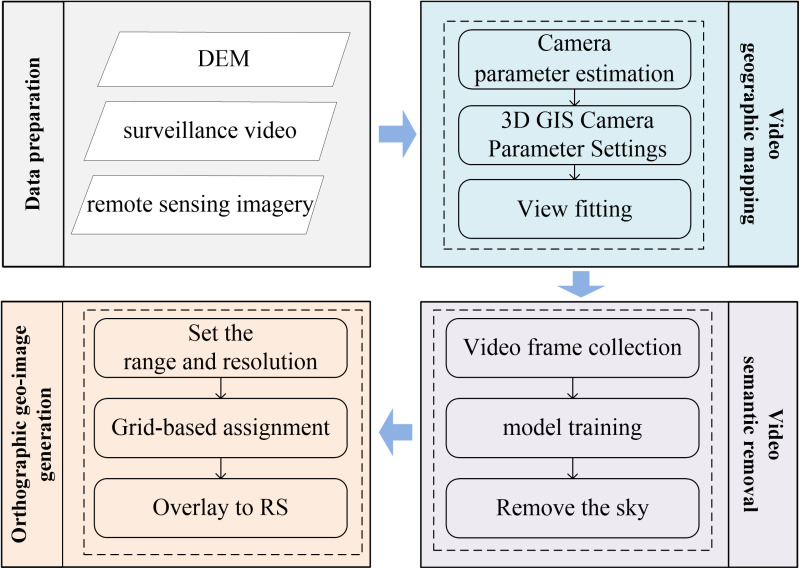
Flowchart of the (ROGI-DS).

Based on real-time PTZ camera video and relying on 2-3D geospatial data [51], this paper proposes a new method of realtime orthographic geo-image generating, which is considering DEM and semantic information (ROGI-DS). It mainly includes three parts: video geographic mapping, video semantic removal, and ortho geo-image generation. The technical process of (ROGI-DS) is shown in Fig 1. The video geographic mapping establishes a mutual mapping model between PTZ video and geospatial space. Video semantic removal, based on a trained semantic segmentation model, automatically removes the sky regions from the video. Orthographic geo-image generation, based on the mutual mapping model and viewshed analysis, calculates the RGB values of the 3D coordinates for each grid in the monitoring area and generates orthographic geo-image.

### 3.1 Video geographic mapping

The video geographic mapping establishes the relationship between the tower video image space and the three-dimensional geographic space, with camera calibration serving as the foundation for this mapping. Tower cameras are widely installed at elevated positions, equipped with 720-degree rotation, zoom functionality, and expansive fields of view. This paper proposes a 3D GIS-based tower camera geographic calibration method (3D GIS-GeoC), which mainly consists of three parts: camera parameter estimation, 3D GIS camera parameter settings, and view fitting.

#### 3.1.1 Camera parameter estimation.

The imaging process of tower video is a mapped from three-dimensional space to image plane two-dimensional space, which follows the camera model. Its core components are the internal and external parameters of the camera. The camera is fixed on the communication tower and generally provides initial location data, including the camera’s longitude (Lon0), latitude (Lat0), and height (H0), but lacks real-time information on azimuth (Azimuth0), tilt (Tilt0), and focal length (f0). Additionally, since the camera’s location data is not determined by professional surveying equipment, there are significant errors. Therefore, tower camera calibration is one of the most crucial techniques in orthographic geo-image generation. Based on DEM, remote sensing images, and structured information of video scenes, this paper proposes a method for estimating the initial values of the camera’s internal and external parameters.

**Fig 2:**
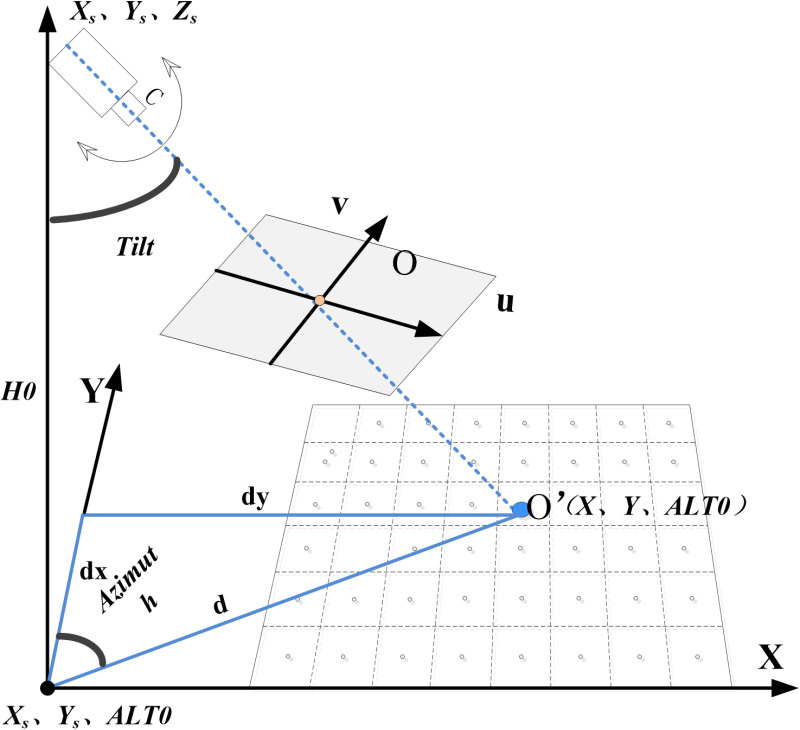
Diagram of camera azimuth and tilt estimation calculation.


First, using the camera’s existing information on Lon0, Lat0, and H0 (H0 is the height of the communication tower), combined with the DEM to obtain the elevation at this location (Alt0), the camera’s geospatial position is Lon0, Lat0, and AH0 (AH0 = Alt0 + H0). To improve the accuracy of the subsequent view fitting, the geographic coordinates (GC) are converted into projected coordinates (PC). The camera’s central position in the projected coordinates is then PC0 (Xs, Ys, Zs). This paper uses Web Mercator map projection. Then, based on the principal point O (u0, v0) of the camera frame, its corresponding geographic location O’ (X, Y, Z) is found in the remote sensing image. The distances between O’ and the camera’s ground point (Xs, Ys) are calculated, including the X-axis distance dx, Y-axis distance dy, and the distance d between the two points. Diagram of camera azimuth and tilt estimation calculation as shown in Fig 2. Given the camera height H0, the Azimuth0 can be calculated using Formula 1, and the Tilt0 can be calculated using Formula 2.

$$Azimuth=arctan\frac{d_{x}}{d_{y}}\backslash )$$ (1)

$$Tilt=arctan\frac{d}{H0}\backslash )$$ (2)

Finally, by selecting two vanishing points in the image (where parallel lines converge), recording their pixel coordinates V1 (u1, v1) and V2 (u2, v2), and using the principal point O (u, v), the initial value of the camera focal length f can be calculated by substituting the vanishing points into Formula 3.


$$\begin{array}{c} \;f=\sqrt{-\left(u_{1}-u_{0}\right)\left(u_{2}-u_{0}\right)-\left(v_{1}-v_{0}\right)\left(v_{2}-v_{0}\right)}\;\backslash \end{array}$$ (3)

#### 3.1.2 3D GIS camera parameter settings.

**Fig 3:**
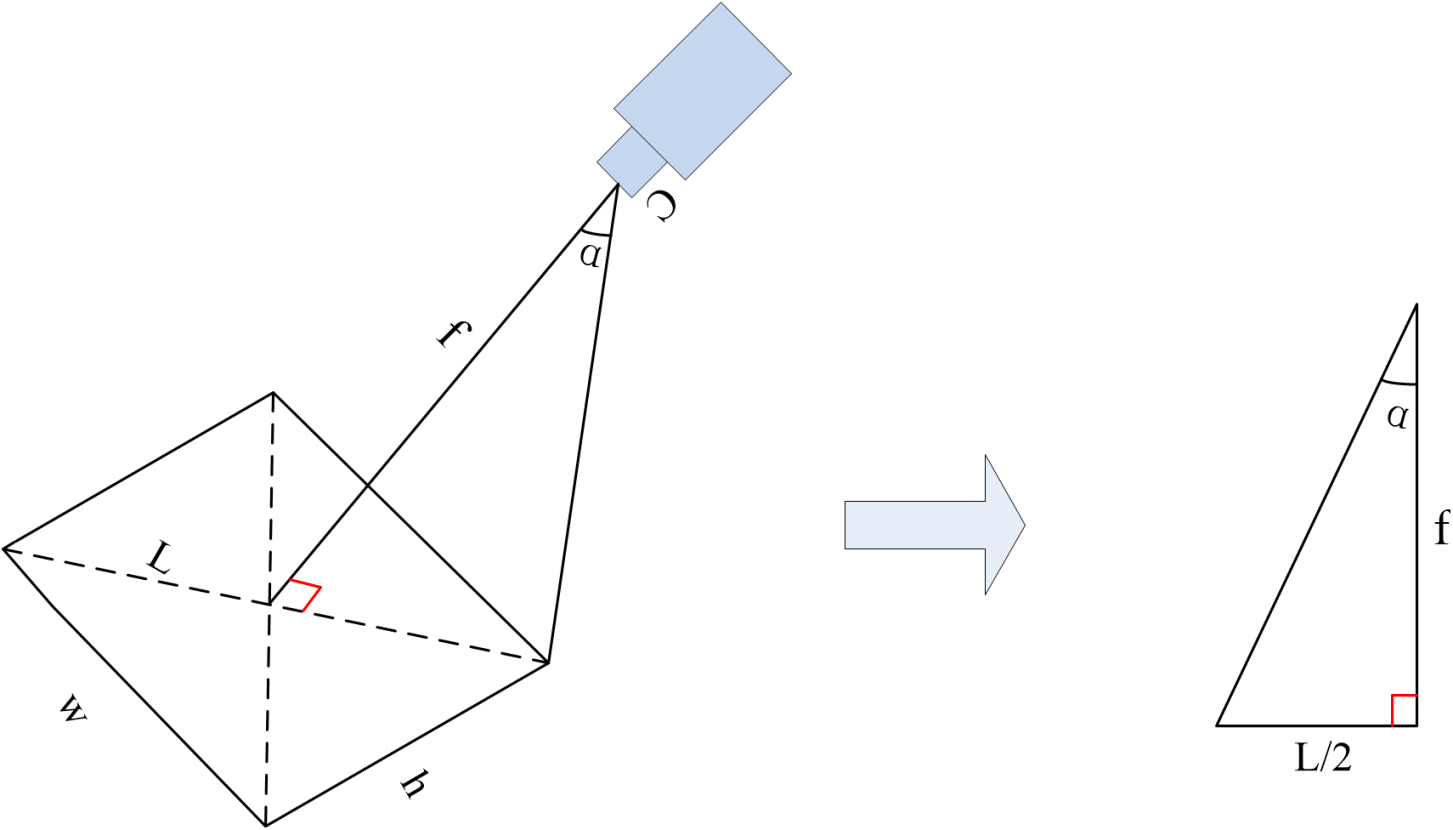
Diagram of FOV calculation in 3D GIS.


3D GIS, which stands for Three-Dimensional Geographic Information System, is an extension of geographic information systems, which represents, analyzes, and visualizes spatial data in a three-dimensional format. To precisely obtain the internal and external parameters of the communication tower camera, this paper converts the estimated camera parameters from Section 3.1.1 to the corresponding parameters for the 3D GIS camera, uses these parameters to configure a 3D GIS camera (which is a observation point). First, convert the external parameters of the communication tower camera (Xs,Ys,Zs). Since the external parameters of the tower camera are the same as those of the 3D GIS camera, therefore, the parameters of the 3D GIS camera remain (Xs,Ys,Zs). Then convert the Azimuth and Tilt of the tower camera. We define the Azimuth, which usually 0 ° in due north direction, increases clockwise, and the range is (0–360 °); We define the Tilt, which is 0 ° perpendicular to the ground, increases upward, and the range is (0–180 °). Due to different 3D GIS platforms may have varying starting positions for Azimuth and Tilt. Therefore, it is necessary to adjust the starting positions and ranges of Azimuth and Tilt on the 3D GIS platform to match our requirements. After adjusting the different 3D GIS platforms, we use the estimated values of Azimuth and Tilt from section 3.1.1 directly as the Azimuth and Tilt for the 3D GIS camera. Finally, the focal length (f) of the tower camera is typically represented by the field of view FOV in 3D GIS camera. Diagram of FOV calculation in 3D GIS is shown in Fig 3. The formula for calculating the diagonal of the image, with frame height Hand width W, is as follows:

$$\begin{array}{c} L=\sqrt{w^{2}+h^{2}}\;\backslash \end{array}$$ (4)

#### 3.1.3 View fitting.

**Fig 4:**
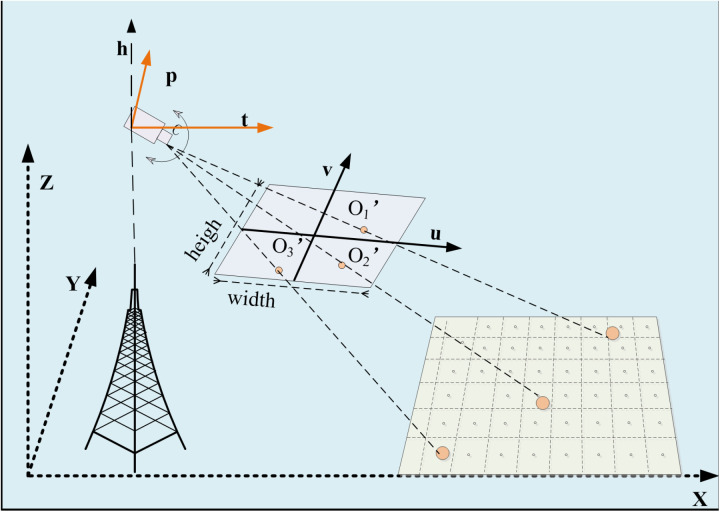
The 3DGIS camera controls the calculation of point pixel coordinates.

**Fig 5:**
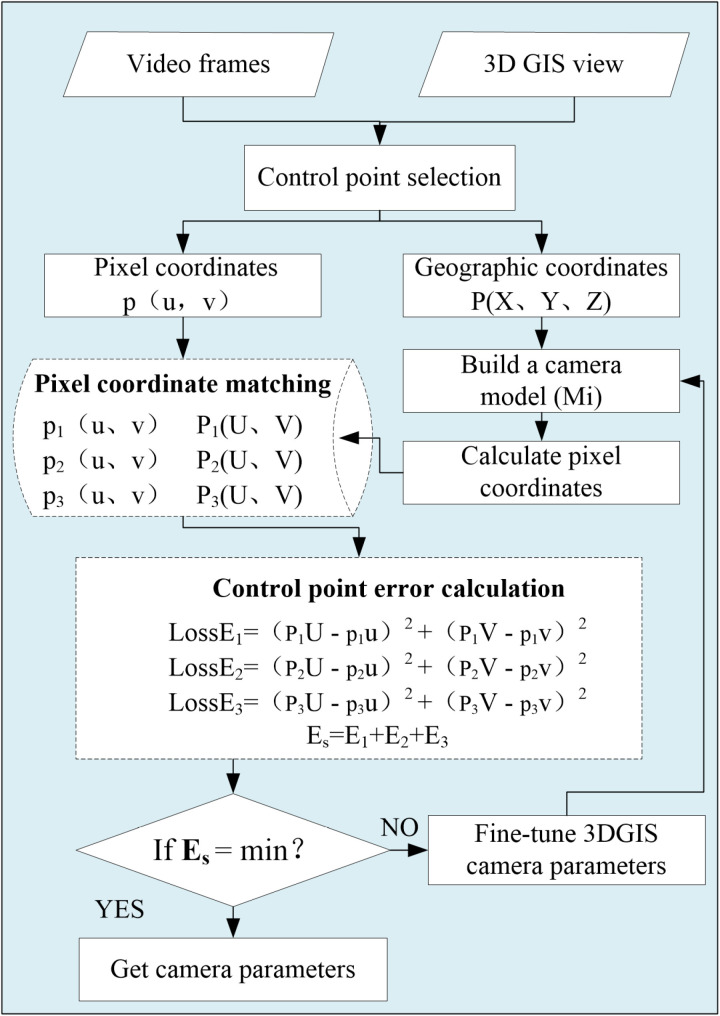
The view fitting flowchart.

However, due to the error of the estimated parameters and the incomplete accuracy of the parameters provided by the tower camera itself, the perspective of the 3D GIS camera is not completely consistent with the perspective of the real tower camera. Therefore, this paper sets a reasonable threshold for each parameter of the 3D GIS camera, namely (RXs = Xs ± 5m, RYs = Ys ± 5m, RZs = Zs ± 5m, RAzimuth = Azimuth ± 10 °, RTilt = Tilt ± 5 °, RFOV = FOV ± 5 °). Then the real parameters of the camera are obtained by view fitting. View Fitting refers to the process of selecting corresponding points (the same points) in the fields of view of two cameras, using these points as control points, and solving for the internal and external parameters of a 3D GIS camera through pixel coordinate matching. Specifically, first, select any object (except the sky) from the tower monitoring video as the control point p of the tower camera, and record the pixel coordinates p_i_ (u, v) of each point, Simultaneously, select corresponding objects in the 3D GIS camera as control points P for the 3D GIS camera, and record the three-dimensional coordinates P_i_ (X, Y, Z) for each point, and calculate the pixel coordinates P′ (U, V) of the control points P′ (X, Y, Z) in the 3D GIS camera using Mi (camera model). The 3DGIS camera controls the calculation of point pixel coordinates as illustrated in Fig 4. Finally, match P′ (U, V) with p (u, v) and calculate the sum of pixel errors of each group of control under the current camera parameters. Then, using an exhaustive search method, fine-tune the parameters of the 3D GIS camera within a specified range, such as（RXs = Xs + 1m、RYs = Ys + 1m、RZs = Zs + 1m、RAzimuth = Azimuth+1°、RTilt = Tilt+1°、RFOV = FOV + 1）. Reconstruct the camera model Mi and use the aforementioned pixel coordinate matching method, again calculate the total error of the control points. Until the total error (Es) of the control points is minimized, record the camera internal and external parameter se t (RXs, RYs, RZs, RAzimuth, RTilt, RFOV). The view fitting flowchart is shown in Fig 5. Use this set as the optimal calibration parameters for the camera. At this point, the fields of view of the 3D GIS camera and the tower camera are synchronized, which creates a one-to-one correspondence between the video image space and the three-dimensional geographic space at the pixel level.

### 3.2. Video semantic removal

Video semantic removal is based on the trained semantic segmentation model to remove the sky areas in the video frame, because the sky does not need to undergo orthographic processing, and which can improve the visualization of the generated orthographic geo-image.

#### 3.2.1 Video frame collection.

To effectively remove the sky area from different viewpoints, this paper constructs a semantic segmentation model (TCSM) for the tower monitoring scene. First, a large amount of real-time and historical video is collected from the experimental area, and video frames were extracted through video decomposition. Then, visual interpretation was used to determine the target categories in the video frames. For categories that cannot be determined through visual interpretation, the camera’s zoom function can be used to closely examine the area and identify category attributes. Finally, different colors were applied for pixel-level manual annotation of each category, producing labeled images corresponding to each video frame. To enhance the model’s generalization capability and prevent overfitting, operations such as rotating and tilting the camera are employed to ensure the diversity of the video frames.

#### 3.2.2 Model training.

Deeplabv3 + is the latest version of the Deeplab series [52]. It builds on DeepLabv3 by adding a decoder module to recover boundary information of semantic objects, and it typically uses classification networks like Res-Net as its backbone [53]. The core part of this model is the Spatial Pyramid Pooling module, which includes a 1 × 1 convolution layer, three 3 × 3 dilated convolution layers, and a global average pooling layer [34]. This paper, based on the Deeplabv3 + network, builds a semantic segmentation model for high-altitude tower surveillance scenes (TCSM)using transfer learning. We label the collected video frame data into six categories: sky, crops, roads, trees, water, and buildings. The data are split into a training set and a test set in a 3:2 ratio. Next, we load a pre-trained network and define the categories within the network, ensuring they match the labeled categories. The network’s parameters are adjusted according to the dataset. Finally, the original images and labeled images are input into the Deeplabv3 + network for training, resulting in a semantic segmentation model (TCSM)for the tower video surveillance scene.

#### 3.2.3 Remove the sky.

**Fig 6:**
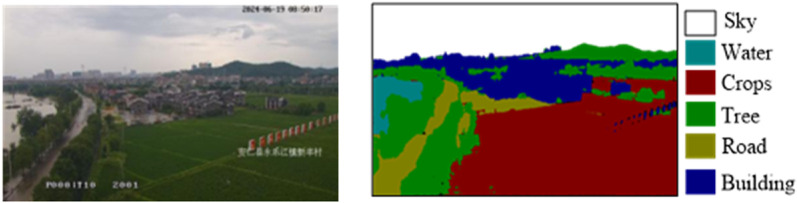
Semantic segmentation results of video frame (Republished from [Fig 6] under a CC BY license, with permission from [Hunan second surveying and mapping institute], original copyright [2024].).

Orthographic geo-image video generation is a surveillance video with orthographic projection. Converting the tower surveillance video into an orthographic geo-image video involves the various ground targets in the video. However, due to the high installation position of the tower camera and its large FOV, the sky is often photographed, this part does not need to generate orthographic geo-image video and should be removed. Additionally, the semantic segmentation model trained in this paper is mainly divided into six categories according to the experimental scenes. Semantic segmentation results of video frame is shown in Fig 6. In this paper, it is only used to remove the sky area, but the (TCSM) can also be applied to further related research areas, such as video change analysis.

### 3.3 Orthographic geo-image generation

Orthographic geo-image generation involves converting tower surveillance videos into orthographic geo-image, which allows for a clear and intuitive understanding of the geographical location and movement direction of targets in the scene. This process mainly includes three parts: set the range and resolution, grid-based assignment, Overlay to RS.

#### 3.3.1 Set the range and resolution.

**Fig 7:**
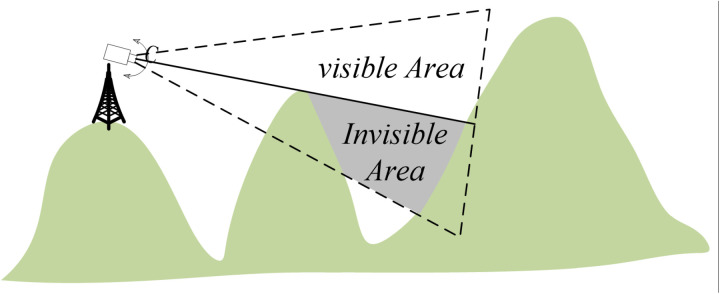
Schematic diagram of the tower camera field of view analysis.

To generate an orthographic geo-image from a single frame of the tower surveillance video, the range and spatial resolution of the orthographic geo-image video must first be determined. The orthographic geo-image range should match the field of view of the tower camera. The field of view of the tower camera refers to the area that can be directly observed from the camera position. However, in the real world, due to terrain effects, there are usually occluded areas. Schematic diagram of the tower camera field of view analysis is shown in Fig 7. where cannot be directly captured by the camera and lack real-time pixel information, should be set as blank.

**Fig 8:**
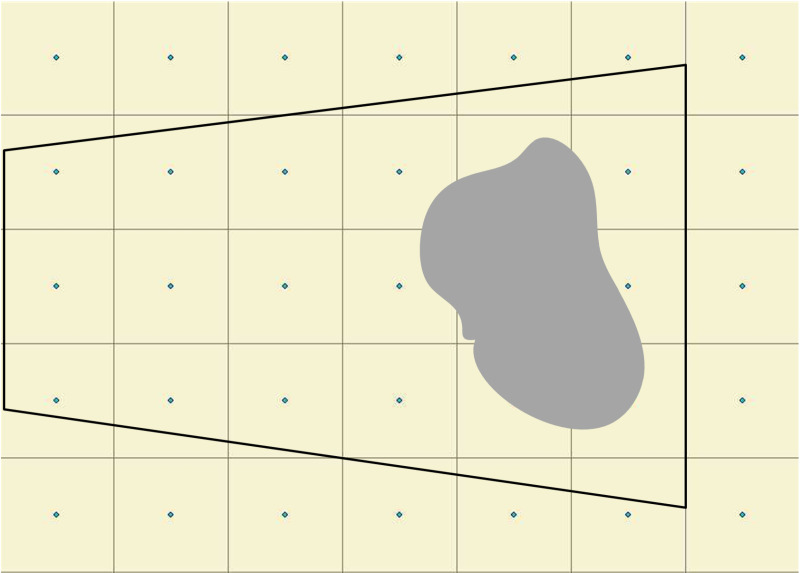
Orthographic geo-image and resolution.

This paper uses camera viewshed analysis to address the issue of terrain occlusion. First, in Arc GIS, the viewshed analysis tool is selected, and based on the camera calibration results from section 3.1.3 (RXs, RYs, RZs, RAzimuth, RTilt, RFOV), the position, azimuth, and tilt angle parameters of the observation point are set. Then, based on the DEM data of the area, the line of sight from the observation point to the target area is calculated, and all potential obstructions are identified. The obstructed areas are removed from the initial field of view, resulting in the actual visible range of the camera’s field of view. Finally, a regular and continuous square grid is created within this range, with the width of each square representing the spatial resolution of the orthographic geo-image. Orthographic geo-image and resolution is shown in Fig 8, the number of grids can be adjusted based on actual needs. The more grids there are, the higher the accuracy of the generated orthographic geo-image and the better the visualization effect.

#### 3.3.2 Grid-based assignment.

**Fig 9:**
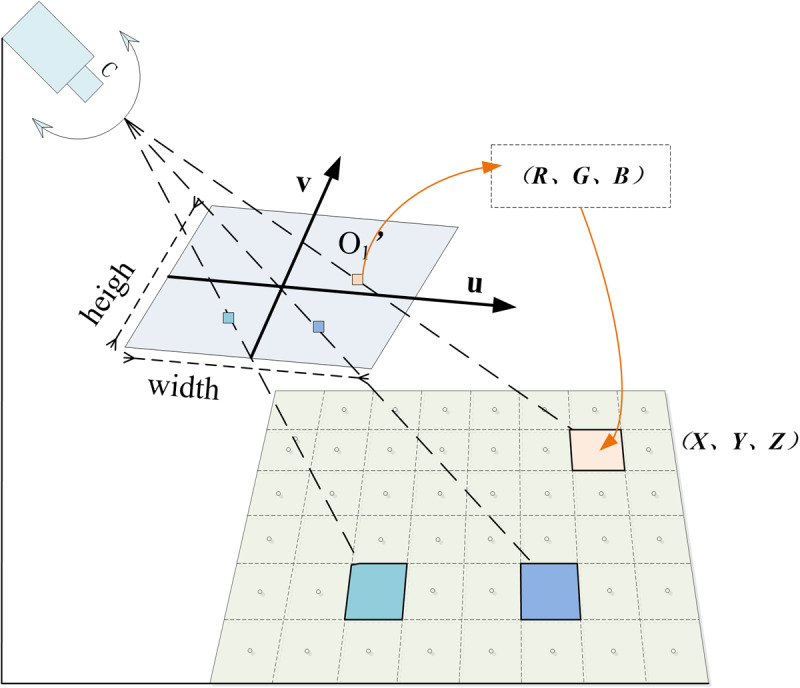
Grid-based assignment diagram.

Grid-based assignment is the process of calculating the corresponding image coordinates through the three-dimensional coordinates of the grid center point, and then assigning the RGB attribute of the image coordinates to the grid. First, based on the orthographic geo-image coverage and the DEM data of the area, the 3D coordinates (X, Y, Z) of each grid’s center point are extracted. Then, based on the camera calibration results, a camera model is constructed. For each grid center Oi (X, Y, Z), the corresponding image coordinates oi (U, V) are calculated, and the RGB values of pixel point o are assigned to grid O. Grid-based assignment diagram is illustrated in Fig 9. In addition, if the DEM variation in a region is minimal (0–3 meters), and the area in the camera’s field of view is a flat plane, this study constrains the DEM value of that region to an average value. This approach not only avoids the influence of DEM accuracy on the results but also improves the visualization of the generated orthographic geo-image.

#### 3.3.3 Overlay to RS.

**Fig 10:**
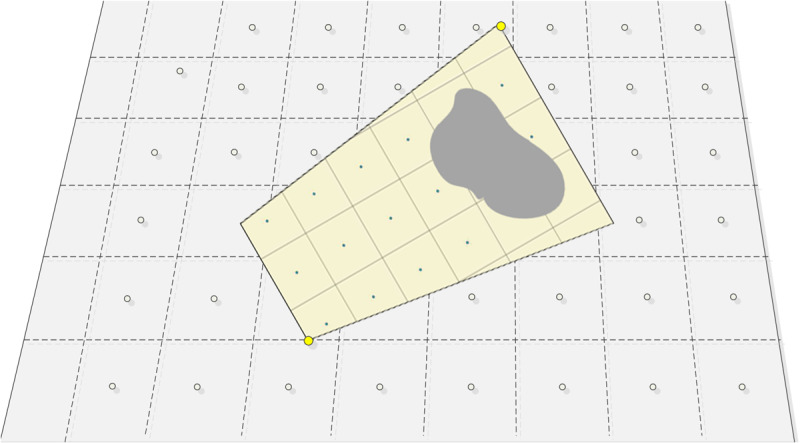
Schematic diagram of JGW coordinate file.

Overlay to RS refers to the process of accurately superimposing the generated orthographic onto the remote sensing image. Specifically, after all grids obtain color attributes, a JPG image and a corresponding JGW format coordinate file are generated based on the grid’s row and column numbers. The JPG image represents the generated orthographic-image, while the JGW coordinate file records the geographic coordinates of the image’s top-left pixel and the geographic coordinate range of the image. The JPW file contains six parameters: the geographic unit distance represented by each pixel in the X and Y directions, and the X and Y coordinates of the top-left and bottom-right pixels of the image. This ensures that the image can be accurately positioned and displayed in GIS. Schematic diagram of JGW coordinate file is shown in Fig 10. After generating each image frame and corresponding coordinate file, these consecutively generated orthographic geo-image frames are integrated and arranged to create the orthographic geo-image.

## 4. Experiments and results

### 4.1 Experimental environment and data

**Fig 11:**
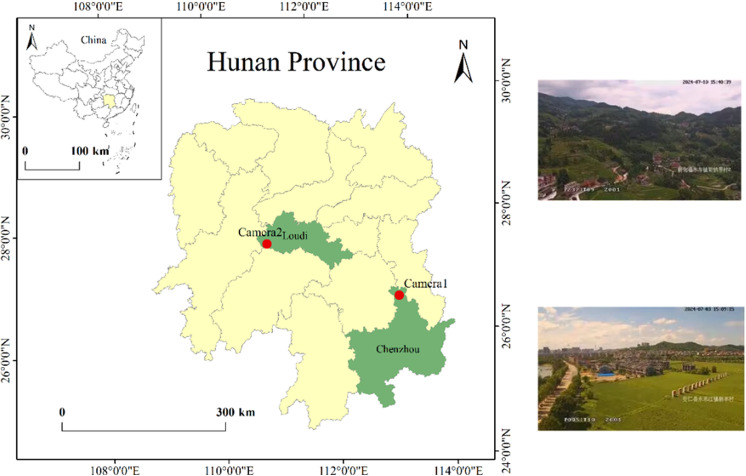
Experimental camera position (Republished from [Fig 11] under a CC BY license, with permission from [Hunan second surveying and mapping institute], original copyright [2024].).

The software environment used in the experiments in this work is VS2012, C#, Python3.6 and Arc-engine, and the hardware environment is a GPU RTX 2080Ti, CPU i9-9820X with 32 GB of memory. In this experiment, we selected two tower cameras located in a region of Hunan Province as examples for the relevant tests. The camera brand is Hikvision, and we used DEM data with a global resolution of 30 meters. Experimental cameras position is shown in Fig 11. Camera 1 is situated in a flat terrain area, while Camera 2 is located in a mountainous area with significant elevation changes. Table 1 presents the parameter information for both cameras. Where lon refers to the longitude of the camera’s location, lat refers to the latitude of the camera’s location, H is the height of the camera in the real world, f is the focal length of the camera, Azimuth is the azimuth angle of the camera, and Tilt is the tilt angle of the camera.

**Table 1:** Experimental camera information.

Parameter	Lon(m)	Lat(m)	H(m)	f(mm)	Azimuth (°)	Tilt (°)
Camera 1	113.281	26.686	122.79	2491	10.30	83.74
Camera 2	110.955	27.717	836.70	2491	38.32	83.74

### 4.2 Experiment analysis

#### 4.2.1 Video geographic mapping.

(1) Camera calibration

**Fig 12:**
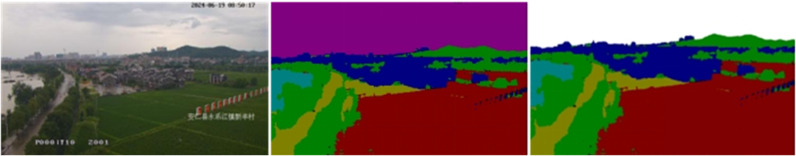
Sky removal result of video frame (Republished from [Fig 12] under a CC BY license, with permission from [Hunan second surveying and mapping institute], original copyright [2024].).

In this paper, camera calibration experiments were conducted on two cameras. First, the initial parameters of the tower cameras were calculated based on the initial external parameters provided by the two cameras and the remote sensing images of the area. These parameters were then converted into 3D GIS camera parameters, and threshold values were set for each parameter.

Then, based on the estimated parameters, we set up a 3D GIS camera. Sky removal result of video frame is show in Fig 12, Due to inaccuracies in our estimated parameters, the views of the tower cameras in both perspectives do not align with the 3D GIS camera. Therefore, we selected eight sets of control points in each perspective and used view fitting to obtain accurate camera parameters. Table 2 and table 3 show the coordinates of the two camera control points

**Table 2:** Control points for camera 1.

	Pixel Coordinates (px)		Geographic Coordinates (m)		
1	668	1443	12610380.312	3084400.108	86
2	1519	1658	12610423.685	3084377.779	87
3	514	1063	12610361.126	3084513.208	84
4	83	861	12610325.409	3084480.191	85
5	696	1104	12610338.206	3084427.973	86
6	2332	1107	12610463.306	3084381.882	84
7	2200	597	12610698.945	3084747.130	85
8	2289	1330	12610414.539	3084363.415	87

**Table 3:** Control points for camera 2.

	Pixel Coordinates (px)		Geographic Coordinates (m)		
1	2341	1298	12351912.820	3213488.950	677
2	506	1391	12351559.831	3213550.271	714
3	1460	820	12351914.964	3213491.996	677
4	709	1287	12351617.745	3213608.239	895
5	395	644	12351637.863	3213793.722	957
6	1901	1278	12351860.470	3213559.248	889
7	1869	650	12352050.535	3213759.253	827
8	722	1270	12351714.744	3213637.658	723

Finally, we calculate the minimum mean square error of pixel matching for the eight sets of control points of the two cameras and determine the optimal internal and external camera parameters, as shown in Table 4

**Table 4:** 3D GIS camera calibration results.

Parameter	Lon(m)	Lat(m)	H(m)	f(mm)	Azimuth (°)	Tilt (°)
Camera 1	113.281065	26.685888	120	2441	11	83
Camera 2	110.955164	27.716409	840	2449	39	84

(2) Comparative Analysis of Camera Calibration Results

To verify the accuracy and reliability of the camera calibration method proposed in this paper, we conducted a comparative analysis with the traditional camera calibration method PnP. We selected 8 sets of control points in the perspectives of both cameras and in the remote sensing images, and recorded their pixel coordinates and geographic coordinates. The distribution and coordinates of the control points are shown below. Then, the DLT(PNP) algorithm was used to calculate the internal and external parameters of the two cameras. we compared the camera parameters calculated using the DLT(PNP) algorithm with those obtained using the 3D GIS-based method and the actual camera parameters, as shown in Table 5. In Table 5, C1 and C2 refer to the two cameras used for the experiment, C1 refers to Camera 1, and C2 refers to Camera 2.

**Table 5:** Comparison of camera calibration results and errors.

	C1	C2	C1	C2	C1	C2	C1	C2	C1	C2
	True value		3D GIS-GeoC		PNP		3D GIS-Error		PNP Error	
Lon(m)	113.281	110.955	113.281065	110.955072	113.281270	110.954892	1.14	11.80	26.43	31.01
Lat(m)	26.686	27.717	26.685888	27.716409	26.685631	27.718983	5.10	9.03	36.73	314.07
H(m)	122.79	836.70	120	840	114.40	747.10	2.79	3.32	8.40	89.61
A (°)	10.30	38.32	11	39	68	98	0.70	0.68	57.70	59.68
T (°)	83.74	83.74	83	84	32.50	15.40	0.74	0.26	51.24	68.34
f(mm)	2491	2491	2441	2449	1422	48	50	42	1069	2443

By comparing the results of two different camera calibration methods for the two experimental cameras, it can be found that the error of the proposed method (3D GIS-GeoC) is smaller, while the error of the traditional method (PNP) is larger. The main reasons include the following aspects: The first is affected by the accuracy of control points. The traditional camera calibration method PNP has high requirements for the accuracy of the control points. However, the tower camera has a relatively broad field of vision. Therefore, when selecting the control points that are far away in the video, it is difficult to obtain their precise three-dimensional coordinates in the remote sensing image, resulting in a large error in the camera calibration results. The camera calibration method (3D GIS-GeoC) proposed in this paper only needs to ensure that the control points are the same ground object, so it is relatively easy and accurate to select the control points with long distance, so its error is small. Second, affected by the distribution of control points, the traditional camera calibration method has high requirements for the distribution of control points, that is, the control points are distributed at different altitudes. However, the tower camera is mainly used to monitor natural resources, and there are a large number of plain areas in its field of view, which is difficult to meet the uniform distribution of control points at different heights. Even in mountainous areas, due to the low accuracy of DEM, it is difficult to select control points or the selected accuracy is low, and it is still unable to obtain accurate three-dimensional coordinates, so it is difficult to calibrate the camera accurately. The camera calibration method based on 3DGIS only needs to ensure that the control points are the same ground object, which is relatively easy and accurate in the selection of control points. Third, due to the influence of camera height difference and the number of control points, the tower camera is installed at a higher position, and the high angle camera will capture more ground information. Therefore, more control points are needed. The camera calibration method based on 3DGIS requires less control points for camera calibration in complex scenes. Therefore, for a large number of tower cameras, the method proposed in this paper can be used to calibrate the camera quickly and obtain accurate camera parameters, which can improve the efficiency of camera calibration. Especially in complex mountainous areas, this method has strong advantages.

#### 4.2.2 Video semantic removal.

The tower camera is installed at a high position, providing a broad field of view, which often includes the sky, and the sky does not need to be orthographic processed. This paper designs a semantic segmentation model for high-altitude tower video scenes, which can automatically remove the sky portion, improving the visual quality of the orthographic geo-image generation. A datasets of 2000 video frames of various scales and different time states is collected in the experimental area to train the tower video semantic segmentation model. The single frame image resolution of tower monitoring video is 2560 × 1440. Since the deeplabv3 + semantic segmentation network uses 512 × 512 images as input by default, this size achieves a good balance between the memory occupation and the computational efficiency, so it is necessary to cut the video frames to adapt to the network input. To meet this requirement, we cut each video image into 512 × 512 small images. Specifically, a video frame with a resolution of 2560 × 1440 can be divided into 15 images with a size of 512 × 512 (5 horizontally and 3 vertically). This can ensure that the original video frame can adapt to the input requirements of deeplabv3 + network, and provide rich training samples for the semantic segmentation model, which improves the performance of the model in the segmentation task. Then labelme software is used to label each small image. Finally, the trimmed small image and the label file are combined to form the data set of the model. In the process of network training, mobilenetv2 is used as the backbone feature extraction network, and the num_classes parameter in the network is adjusted to be consistent with our annotation category. In order to obtain more details of the image, set the down-sample factor to 8, set the ratio of the training set and the verification set to 9:1, and set the number of training rounds to 100. The software environment for training is pytorch 1.13.1 + cu116, and the hardware environment is a GPU RTX 3050ti, CPU i5-12500H with 32 GB of memory. The training duration is 55 minutes, with a test set accuracy of 98.70%. The semantic segmentation effect is shown in Fig 12.

#### 4.2.3 Orthographic geo-image generation and overlay.

**Fig 13:**
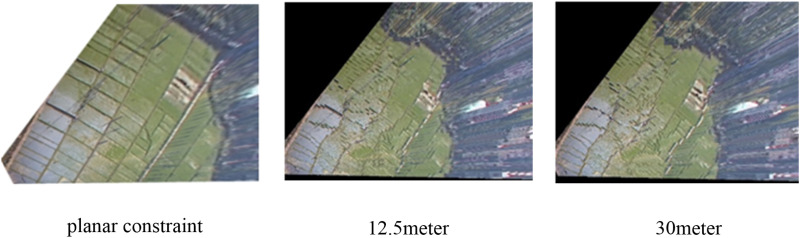
Orthographic geo-image generation of camera 1.

**Fig 14:**
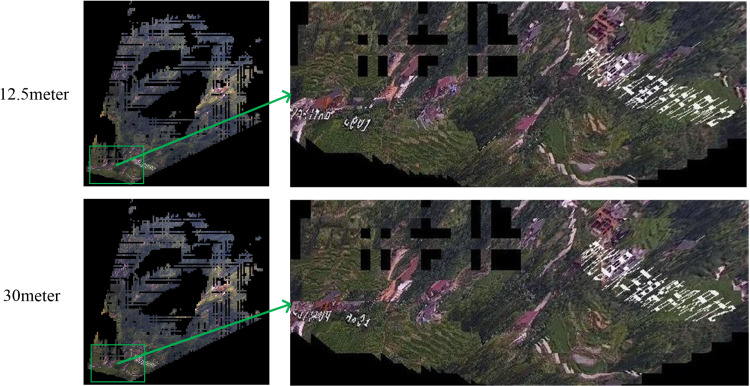
Orthographic geo-image generation of camera 2.

To analyze the visual effects of orthographic geo-image generation under different terrain conditions, this study conducts orthographic geo-image video generation experiments from two perspectives for each of the two cameras. Based on the real-time videos captured by the two cameras, the actual field of view of the cameras is delineated in the remote sensing images. Camera 1 is located in a plain area with a smaller field of view, whereas Camera 2 is situated in a mountainous area with a broader field of view. We set the orthographic geo-image resolution for both cameras to 1 meter (each grid cell is 1 meter by 1 meter). Since the 5-meter and 10-meter DEM data are not yet public, this experiment selects 12.5-meter, 30-meter, and elevation constraint DEM data to generate orthographic geo-images and makes a comparative analysis of the experimental results. Fig 13 and Fig 14 show the effect comparison of DEM with different resolutions in generating orthographic geo-images. For plain areas, the orthographic geo-images generated by 30-meter resolution DEM show significant deformation, especially at the boundary of roads and farmland, with low spatial accuracy. In contrast, the visualization effect of orthographic geo-images generated by 12.5-meter-resolution DEM has improved. Although there is still a certain degree of boundary dislocation, the overall accuracy is better than that of the 30-meter-resolution DEM. Additionally, since Camera 1’s field of view is in a flat area and the DEM data used in this study has a resolution of 30 meters, we constrain the elevation of the area to an average value of 84 meters to avoid visual impact from the DEM data’s precision. The orthographic geo-images generated by the DEM elevation constraint method maintain the integrity of the road and cultivated land boundary within the field of view, showing the best visualization effect. Therefore, in the plain area, when there is a lack of high-resolution DEM data, the DEM elevation constraint method can effectively improve the visualization quality of orthographic geo-images. For mountainous areas, it can be found that the visualization effect of DEM data with 30-meter resolution is poor, and the roads and other areas in its field of view are severely deformed. The visualization effect of DEM data with 12.5-meter resolution is relatively good, and the visualization effect of roads and other areas in its field of view is better, especially in areas with large elevation changes. Due to the complex terrain in mountainous areas, the elevation constraint method is difficult to achieve accurate constraints on the real elevation of each point. Therefore, higher resolution DEM data can be used to obtain orthographic geo-images with higher accuracy and better visualization effect.

By analyzing the experimental results of the two cameras, it can be seen that the (ROGI-DS) in this paper has a good visualization effect in the two experimental sites. Since Camera 1’s field of view mainly covers flat terrain, the resulting orthographic geo-image exhibits good visualization quality. Camera 2, located in a mountainous area with significant elevation changes, has areas of mutual occlusion. The (ROGI-DS) method removes the invisible areas after analyzing the field of view, resulting in overall good visualization quality. It effectively addresses the problem of severe distortion in areas with significant elevation changes in the video, significantly enhancing the visualization and measurability of the orthographic geo-image.

To better analyze the advantages of this method, we conducted a comparative experiment with another orthographic video generation method that combines video and GIS — the homography matrix method. The homography matrix method uses the perspective transformation relationship between two images to achieve mapping between them.

**Fig 15:**
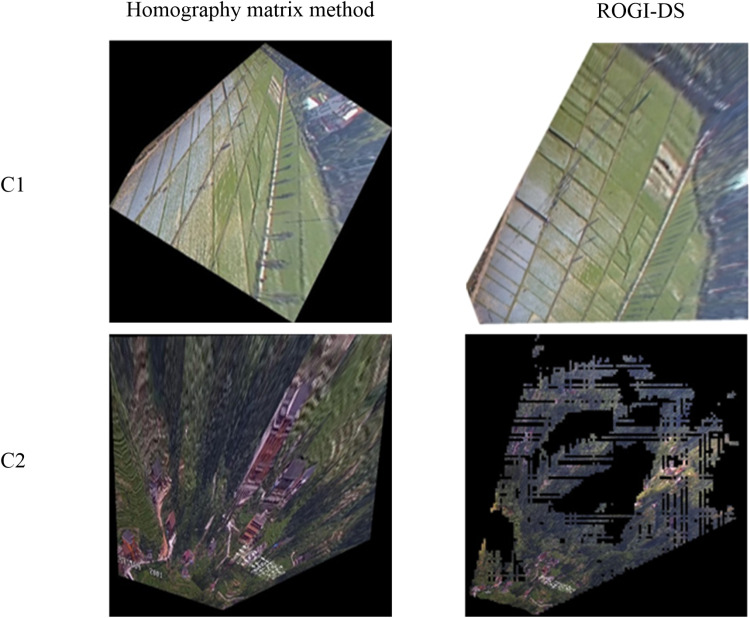
Comparison of different methods for generating orthographic geo-image.

First, in the experimental scenario, we selected four sets of control points to compute the homography matrix between two images. Then, using this homography matrix, we generated two orthographic geo-images, as shown in Fig 15. By comparing the experimental results of both methods, we found that the ROGI-DS method proposed in this paper outperforms the homography matrix method in terms of visualization effects, both in mountainous and plain areas. Particularly in mountainous regions, the orthographic geo-images generated by the traditional homography matrix method show poor accuracy and visualization effects. This is because the homography matrix method is only suitable for flat areas and requires high precision in the control points. In mountainous regions, the orthographic geo-images generated by this method fail to achieve good visualization. In contrast, the method proposed in this paper, based on the camera model and processes such as semantic segmentation and viewshed analysis, improves both the accuracy and visualization effects of the orthographic geo-images.

## 5. Discussion

In this paper, a geographic calibration method for tower cameras is proposed based on tower cameras and 3D GIS. Based on the calibration results, an orthographic geo-image generation method considering DEM and semantic information is designed. Compared with the traditional camera calibration method, the method proposed in this paper can obtain the camera parameters more accurately, especially for the high-precision calibration of large-scale tower cameras. Based on the calibration results, the orthographic geo-image generation method designed in this paper has better visualization effects. The traditional camera calibration method has high requirements for the distribution and accuracy of control points, while the tower camera often requires extensive fieldwork to obtain accurate control point coordinates due to its wide field of view and complex scene. The geographic calibration method for tower cameras based on 3D GIS proposed in this paper only needs to ensure that the control points fall on the same pixel, and accurate camera parameters can be obtained through camera model fitting. At the same time, whether the camera is located in a plain or mountainous area, this method can generate orthographic geo-images with better visualization effects compared to traditional methods, with the effect in the plain area being significantly better than that in the mountainous area. The study also found that with the improvement of DEM accuracy, the visualization effect of orthographic geo-images was further optimized; if the camera is located in plain areas, the use of elevation constraints can significantly improve the visualization effect of orthographic geo-images. This method is not only applicable to the monitoring of natural resources but can also provide orthographic geo-image backgrounds for parks, central squares, and other planar areas in urban environments. Combined with target detection technology, crowd information can be projected onto the map to assist in the efficient implementation of security work.

Although this method provides better visualization effects than traditional methods, it still has some limitations: the current generation time for each orthographic geo-image is approximately 20 minutes. To meet the requirements of real-time applications, grid partitioning, time-based generation, and improved computing performance can be used to accelerate the generation speed. In complex mountainous and urban building overlap areas, the visualization effect of the orthographic geo-images generated is not ideal due to the limitations of camera side-view imaging. In future research, this method can be combined with optimization measures for planar areas; for mountainous and urban building overlap areas, we can attempt to integrate deep learning technologies, use monocular depth recovery methods, and combine them with the techniques presented in this paper to generate three-dimensional maps, improving the visualization effect of orthographic geo-images in mountainous areas. In conclusion, this paper proposes an effective geographic calibration method for tower cameras based on 3D GIS and an orthographic geo-image generation method, which significantly improves the visualization effect of orthographic geo-image generation. Although there is still room for improvement in generation speed and complex scene processing, this method shows strong application potential in natural resource monitoring and urban environmental safety, laying the foundation for further optimization and expansion.

## 6. Conclusions

Tower cameras, with their extensive coverage and elevated perspective, are increasingly becoming new tools for the management and protection of natural resources. Therefore, exploring the spatialization of tower video is of significant importance. A mapping model between the tower video image space and the 3D geographic space is established. this paper proposes a new method of realtime orthographic geo-image generating, which is considering DEM and semantic information ROGI-DS.

The experimental results show that: (1) Compared with traditional methods, the 3D GIS-GeoC method can automatic and efficient calibration of tower cameras, thereby improving both the efficiency and accuracy of tower camera calibration. (2) This paper trained a semantic segmentation model for tower surveillance video scenes, which can accurately remove the sky area in the surveillance scene, thus improving the efficiency of orthographic geo-image generation. Additionally, the model can precisely classify different types of objects. (3) To address the deformation issues in non-planar regions (such as mountainous areas, ROGI-DS method can improve the accuracy and visualization of orthographic geo-image c video under different terrain constraints.

Of course, although the orthographic geo-image generation method proposed in this paper improves the visualization effect of orthographic geo-images, the visualization effect of orthographic geo-images generated in mountainous areas is poorer than that in flat areas. Therefore, in future research, we will attempt to use monocular depth recovery technology combined with this method to create side-view maps and 3D maps with higher expressive power for mountainous areas, thereby overcoming the limitations of current methods in mountainous terrain. At the same time, the tower camera has rotation and zoom functions. When the camera rotates, the generation and fusion of orthographic geo-images from other perspectives will also be a key focus of our future research.

Due to the large number of abbreviations used in this article, we will show them in Table 6 for ease of understanding.

**Table 6:** Abbreviations and their full names.

PTZ	Pan-Tilt-Zoom
DEM	Digital Elevation Model
ROGI-DS	A new method of realtime orthographic geo-image generating, which is considering DEM and semantic information.
3D GIS-GeoC	3D GIS-based tower camera geographic calibration method
TCSM	Tower Camera Semantic Segmentation Model
PnP	Perspective-n-Point
ICT	Information and communication technology
2D/3D	Two/Three-Dimensional Map
video GIS	Video Geographic Information System
Lon	Longitude
Lat	Latitude
H	Height
f	Focal length
FOV	Field of View
GC	Geographic Coordinates
PC	Projected Coordinates
h	Image height
w	Image width
L	Image diagonal length
RS	Remote Sensing Image
DLT	Direct Linear Transformation
